# Increasing Leaf Vein Density by Mutagenesis: Laying the Foundations for C_4_ Rice

**DOI:** 10.1371/journal.pone.0094947

**Published:** 2014-04-23

**Authors:** Aryo B. Feldman, Erik H. Murchie, Hei Leung, Marietta Baraoidan, Robert Coe, Su-May Yu, Shuen-Fang Lo, William P. Quick

**Affiliations:** 1 School of Biosciences, University of Nottingham Malaysia Campus, Semenyih, Selangor Darul Ehsan, Malaysia; 2 School of Biosciences, University of Nottingham Sutton Bonington Campus, Sutton Bonington, Leicestershire, United Kingdom; 3 Plant Breeding, Genetics and Biotechnology, the International Rice Research Institute, Los Baños, Philippines; 4 The C4 Rice Center, the International Rice Research Institute, Los Baños, Philippines; 5 Institute of Molecular Biology, Academia Sinica, Nankang, Taipei, Taiwan; National Taiwan University, Taiwan

## Abstract

A high leaf vein density is both an essential feature of C_4_ photosynthesis and a foundation trait to C_4_ evolution, ensuring the optimal proportion and proximity of mesophyll and bundle sheath cells for permitting the rapid exchange of photosynthates. Two rice mutant populations, a deletion mutant library with a cv. IR64 background (12,470 lines) and a T-DNA insertion mutant library with a cv. Tainung 67 background (10,830 lines), were screened for increases in vein density. A high throughput method with handheld microscopes was developed and its accuracy was supported by more rigorous microscopy analysis. Eight lines with significantly increased leaf vein densities were identified to be used as genetic stock for the global C_4_ Rice Consortium. The candidate population was shown to include both shared and independent mutations and so more than one gene controlled the high vein density phenotype. The high vein density trait was found to be linked to a narrow leaf width trait but the linkage was incomplete. The more genetically robust narrow leaf width trait was proposed to be used as a reliable phenotypic marker for finding high vein density variants in rice in future screens.

## Introduction

The yield potential of rice (*Oryza sativa* L.) needs to be increased by at least 50% by 2050 to support the burgeoning human population, which can only be underpinned by improvements in rates of biomass production [1]. Rice productivity could be radically improved by the introduction of C_4_ photosynthetic properties. C_4_ photosynthesis is characterized by a CO_2_ concentrating mechanism involving the coordination of metabolism in two cell types, the mesophyll and bundle sheath. It results in the elimination or substantial reduction in photorespiration and consequently an enhancement in the capacity and quantum yield of photosynthesis at high temperatures [2] [3] [4].

One of the key C_4_ properties is a high leaf vein density, which has been considered to be a prerequisite to the evolution of the complete suite of C_4_ traits in plants [5] [6] [7]. It is needed to ensure the optimal ratio of mesophyll and bundle sheath cells with close contact permitting the rapid exchange of photosynthates. This is achieved via ‘Kranz’ anatomy which typically shows a single or double layer of mesophyll cells enclosing bundle sheath cells in a concentric fashion. Bundle sheath cells in turn enclose the vascular tissue. This arrangement permits bundle sheath cells and mesophyll cells to occupy similar volumes within the C_4_ leaf, whereas the total mesophyll cell volume is greater in the C_3_ leaf [8]. A possible source of study for identification of rice genes regulating traits relevant to C_4_, like high leaf vein density, is mutagenized populations.

Chemical and physical deletion mutagenesis is an important tool in forward and reverse genetics, in addition to inducing new traits and enabling various allelic expressions of certain genes by turning off suppressor genes. Point mutations, caused by EMS mutagens for example, are the removal of a single base pair in the DNA molecule. A more sizeable amount of DNA can be removed by larger deletions. For example, γ-ray mutagenesis can delete a region from 1–4 kb in size. Deletion mutagenesis is especially useful as it can produce phenotypic variation representing alterations to the entire genome of any genotype in a relatively efficient fashion [9] [10]. The value of deletion mutagenesis for rice breeding is reflected by the size of the stock of cv. IR64 mutants held at the International Rice Research Institute (IRRI): 66,891 M_4_ lines [10]. Mutagenesis has been widely used in crop improvement. The most successful mutation in rice breeding has been the *sd1* gene, which reduces plant height by 25% without affecting panicle size to diminish lodging. The gene was a spontaneous monogenic recessive mutation and is now present in over 90% of modern high yielding varieties [11]. Other important traits in rice breeding programs induced by mutagenesis include early flowering, low phytic acid content and giant embryo size [12]. The IR64 mutant library itself has been a source of resistance to diseases [13] [14] [15], high biomass production [Barroiden M, IRRI, personal communication], salt tolerance [16] and drought tolerance [17], as well as the identification of genes involved in leaf development [18].

Deletion mutagenesis has an advantage over other means of inducing new traits as deletions usually knockout multiple genes. This is relevant to developing a C_4_ rice as it is possible that more than one gene copy in rice would need to be removed to ensure the loss of expression and to discover gene function [19]. Deletion mutagenesis is also able to induce mutations at high rates and so can produce a set of genome wide saturated mutations in a relatively small population [20].

Insertion mutagenesis is designed to disrupt and/or activate gene expression while tagging the site of insertion. The inserted fragment of DNA can inactivate a gene if it is located in the middle of its coding sequence. The insertional construct may also contain activation vectors that carry strong promoters, which can function in either orientation and at a considerable distance from the coding regions. These can cause transcriptional activation of nearby genes, resulting in dominant gain of function mutations [21]. It has been shown that activation tags have an effect of up to 10 kb left and right of the insertion point [10], so can affect multiple genes by individual insertion events. Insertional mutagenesis includes a variety of insertion elements such as T-DNA, transposable elements like the *Tos17* retrotranspon, and the Ac-Ds and Spm/dSpm maize transposons. The Taiwan Rice Insertion Mutant (TRIM) library at Academia Sinica in Taiwan holds 100,000 T-DNA lines with a cv. Tainung 67 background [22].

Insertion mutagenesis is relevant to engineering a C_4_ rice since the different attributes of C_4_ photosynthesis include both the addition and removal of biochemical and anatomical features found in C_3_ plants. Tagged insertion mutants are of special interest because they can represent ‘engineered’ expression levels of known gene sites, so that correlations between tags and phenotypes can be made. The disadvantage of deletion mutagenesis is that it can produce silent mutations, i.e. changes in the base sequence that have no mutant phenotype (due to gene redundancy, for example). Therefore, by enhancing the expression of genes near the insert in insertional mutagenesis, novel variation may be generated in the C_3_ genome that is relevant to C_4_ photosynthesis.

Alterations in gene function by mutagenesis of rice can be explored for phenotypic changes relevant to C_4_ photosynthesis. The success of this relies upon the hypothesis that the rice genome has sufficient functional polymorphism and genetic plasticity to yield measurable C_4_ traits that can be revealed by artificially inducing variation in the genome. Mutagenesis of rice provides a source of genetic variation that can cover the entire genome and so can give a good indication of whether this is the case. Following a forward genetics scheme allows the detection of relevant phenotypes whose underlying genetics can be characterized.

This paper takes such an approach and describes the high throughput screening of large numbers of lines within rice mutant populations for leaf vein density and the subsequent genetic analysis of resulting high vein density candidates. Here we conduct a study to test the hypothesis that vein density is a heritable trait in rice and that mutant populations can be used to generate phenotypic variation for the study of traits in rice that are relevant to C_4_ photosynthesis. We also investigated the close link of leaf vein density with leaf width, which was found in the high vein density mutant lines that had narrow leaf widths in [23]. The possibility of uncoupling the two traits was investigated by elucidating the genetic bases of leaf width and vein density in rice mutants.

## Materials and Methods

### Vein density screening

The primary genetic resource was the IRRI IR64 deletion mutant population derived from a single IR64 plant, IR64-21 [13]. The original IR64-21 parent plant was selected for phenotypic uniformity from breeder seeds grown under field conditions. IR64 mutants used in the screening were either deletions (mainly dry seeds mutagenized with γ-radiation at 250 GY at the International Atomic Energy Agency) or point mutations (mainly pre-soaked seed mutagenized with EMS at 0.4, 0.6, 0.8, 1.0 and 1.6% concentrations at IRRI). Mutagenized lines were advanced every four months from the initial M_1_ population by single seed descent until at least the M_3_ generation. This was to ensure near homozygous inbred lines, which was essential for quantifying phenotypic characteristics in replicated trials. The screened population, consisting of 12,470 lines, was at the M_4_ generation derived from either bulked sibling seeds or single seed descent. The deletion mutant population was divided by leaf width phenotypes: wild-type and narrow leaf width. The first vein density screen included only those mutants with wild-type leaf widths whereas the second screen targeted narrow leaf width mutant lines.

The secondary mutant population screened for variations in vein density was 10,830 insertion lines from the TRIM library, produced by Academia Sinica. The TRIM library consisted of lines that had gene disruptions or activation tags by use of randomly inserted *Agrobacterium tumefaciens* mediated transfer DNA (T-DNA) (average copy number of 1.7) [24].

Ten seeds per line were planted in the deletion mutant vein density screening. However, germination rates were rarely 100% and plants with gross morphologies (e.g. those that were severely pale or stunted) were disregarded for screening. Thus, generally 5–8 plants per line were sampled for screening. Ten wild-type IR64 seeds were planted as controls alongside the planting of mutant plants for every day of measurements (per 170 lines). Veins were counted on-site using a handheld microscope (Readiview handheld microscope, Meade Instruments Corporation, Irvine, USA) at 80× magnification.

A similar number of plants per line were sampled in the insertion mutant population. Here, vein counts were made off-site at IRRI from images that were taken of freshly harvested material. Images of veins in the freshly harvested youngest fully expanded leaf at the fifth to the seventh leaf stage were captured for screening the T_1_ insertion mutant population by a small digital microscope (AM7013MT Dino-Lite Premier, AnMo Electronics Corporation, Torrance, California, USA) at 4× magnification. Imaged veins in four × 2 mm wide areas of the leaf (not including the midrib or edges) were counted.

The fifth to the seventh fully developed leaf that emerged on the primary tiller, excluding the seed leaf (leaf five to seven), was used for vein density measurements in both populations of mutants. The number of veins in the widest part of the abaxial side of the leaf was counted. It was important to take care to always count the veins of the widest middle part of the leaf as some vein density variation was observed towards the the base and tip (unpublished observations). Care was also taken to avoid counting veins close to the midrib and close to the margins of the leaves, where there was a tendency for vein density values to increase and so they were not representative of the entire leaf width (unpublished observations). If a high vein density value was recorded, then a further vein density reading was taken in the leaf area of the other side of the midrib. If two consistently high vein densities were found and this was confirmed by microscopy analysis, the plant was considered to be a candidate.

Potential candidates detected from the vein density screening had their vein densities confirmed using a laboratory microscope equipped for digital imaging. Vein numbers of fresh leaf samples were viewed with a light microscope (Olympus BX51 Motorized Research Microscope, Tokyo, Japan, connected to an Olympus DP71 Microscope Digital Camera, Tokyo, Japan) at 4× magnification. Images were taken using Olympus Cell∧P imaging software (from the Olympus Cell* software family).

#### Crossing

Deletion mutant lines were backcrossed to their parental line, IR64-21, to remove a significant portion of background mutations in an effort to revert all traits to wild-type except for high vein density. Mutations were assumed to be recessive, given their low level of detection and the nature of chemical and physical mutations [Hei Leung, IRRI, personal communication], and so were designated as the female parent. This made the recognition of true BC_1_F_1_ plants easier as they should have exhibited the wild-type phenotype.

Mutant plants that had begun heading were emasculated in the afternoon (at ∼15:00). All spikelets (palea and lemma) on the panicles were cut at an obtuse angle (>45°) with small scissors so that the anthers were exposed and the stigma was left unharmed. Anthers were removed with a vacuum emasculator (Gast Model-1022-V103-G272X, Gast Manufacturing Corporation, Benton Harbor, Michigan, USA). Their panicles were then bagged to make sure that stigmas did not desiccate or were unintentionally fertilized.

The following morning (at ∼10:00), after removing the bags from the panicles, forceps were used to remove any remaining anthers if there were any. Remaining naked stigmas were then fertilized with the parent plant, whose anthers protruded outside the spikelet. Pollen was dusted onto the receptive female by tapping the stems of the pollen donor panicles beside the emasculated spikelets. Pollen dusting was repeated the following two mornings to ensure successful pollination. Panicles were then bagged for seed setting.

### Growing conditions

The initial T_1_ insertion mutant population was grown under field conditions at Academia Sinica (25° 4′ 34.51′′ N; 121° 61′ 44.91′′ E), Taiwan and screened in the summer 2009 (Oct. 2009). The mean temperature was 28.8°C (range: 19.4–38.6°C), mean relative humidity was 74.3%, and mean daily sunshine duration was 5.0 hours.

Subsequent analysis of the insertion mutant population and of the deletion mutant population took place at the IRRI greenhouses (14° 10' 23.29", 121° 15' 32.44") from mid-2009 to mid-2012. The mean temperature was 26.8°C (range: 19.9–35.0°C), mean relative humidity was 86.3%, and mean daily sunshine duration was 6.4 hours.

Seeds were incubated at 50°C for 72 hr if their dormancy had to be broken, i.e. if seeds were more than two years old or if germination rates were particularly poor (<30%). They were then pre-germinated in petri dishes on filter paper treated with sterilized distilled water and incubated at 35°C until the radical and coleoptile were sufficiently swollen (usually by 24 hr).

In the screening of deletion mutants, 100 seeds were planted in a tray with length width x height dimensions, 36.5 cm × 27.0 cm × 11.5 cm. For both rice populations, soil was sourced from the IRRI upland farm and was treated with 0.09-0.01-0.09 g NPK fertilizer per kg soil as a basal dressing and 0.09 g N fertilizer per kg soil every 2–3 weeks, depending on plant growth rate (determined qualitatively on a relative scale). Irrigation was applied generously to ensure that the soil moisture content was always high.

## Results

### Vein density screening

**Figure 1:**
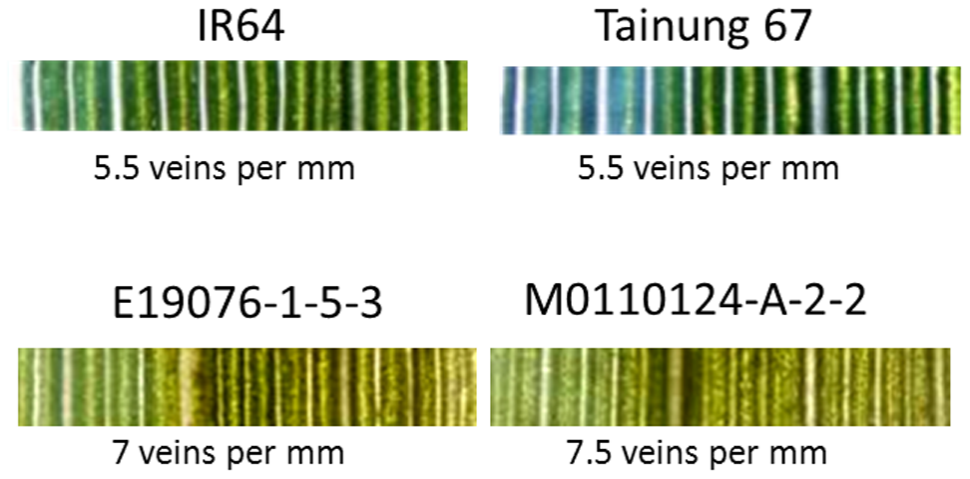
Leaf veins of mutant and wild-type rice lines. Images of veins within a 2 mm leaf width were captured at 2–3 weeks after sowing. Line E19076-1-5-3 is an M_5_ IR64 mutant. Line M0110124-A-2-2 is a T_3_ Tainung 67 mutant.

**Figure 2:**
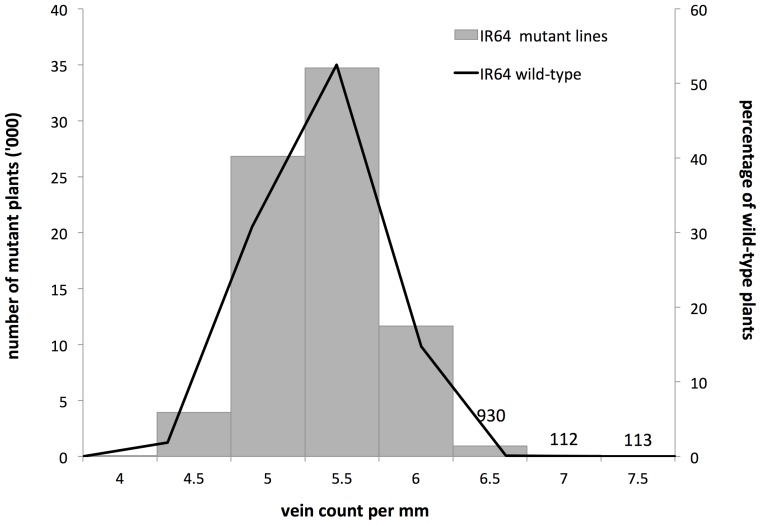
Frequency distribution of vein densities in the deletion mutant population and their control in the screen. Values above chart bars denote the number of mutant plants for each high vein density. Distribution frequencies of deletion mutant and control plants were largely the same. Differences were at the extremity of the binomial curve at higher vein densities (930 (1.2%), 112 (0.14%) and 13 (0.02%) mutant plants for respectively 6.5, 7 and 7.5 veins mm^−1^) where lines were selected as potential candidate mutants for increased vein density.

Vein densities of the IR64 wild-type were largely (83%) 5 or 5.5 veins mm^−1^ (Figures 1 and 2). They were rarely (0.10%) 6.5 veins mm^−1^ and those instances were confined to one side of the midrib only. In addition, the average vein density of all deletion mutant plants was 5.4 veins mm^−1^, which was more than two standard deviations (i.e. >0.7) away from 6.5 veins mm^−1^. Hence, any plant that had a vein density of 6.5 veins mm^−1^ or more on both sides of the midrib was considered to be a potential high vein density candidate.

Figure 2 shows that there were overlapping frequency distributions of vein density values of the IR64 wild-type line and M_4_ deletion mutant plants for vein counts per mm. This meant that mutagenesis did not affect the vein density of most of the lines. Those that showed differences in vein density were at the upper tail end of the bell shaped curve.

**Figure 3:**
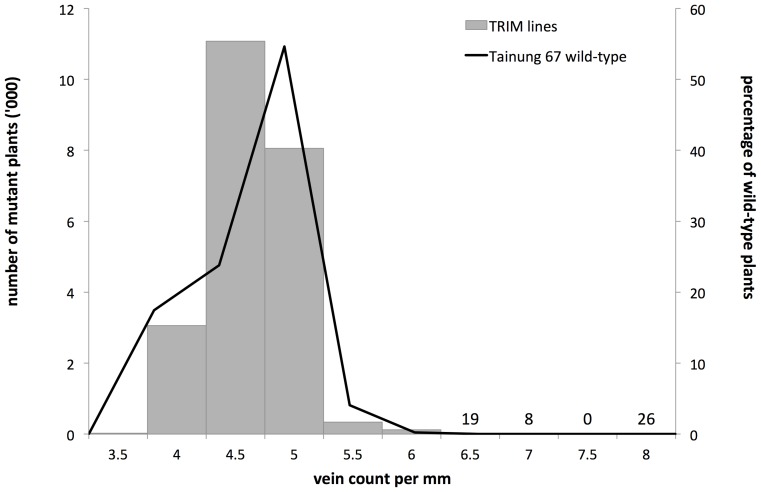
Frequency distribution of vein densities in the T_1_–T_3_ insertion mutant populations and their controls. Values within the chart denote the number of mutant plants for each of the highest vein density values. Distribution frequencies of insertion mutant and wild-type plants were largely the same. Differences were at the extremity of the binomial curve at higher vein densities (eight (0.04%) and 26 (0.12%) mutant plants for respectively 7 and 8 veins mm^−1^) where lines were selected as potential candidate mutants for altered vein spacing.

The screening of 10,830 T_1_ insertion lines showed that leaf vein density frequencies of the Tainung 67 wild-type and insertional mutants were similarly distributed (Figure 3). As was the case in the deletion mutants (Figure 2), this implied that mutagenesis did not affect the vein density in the large majority of insertional lines but rather created enough variation to produce a small fraction of mutants with an increase in vein density. The distribution frequency of insertion lines had a heavier tail compared to the Poisson distribution of the deletion mutants.

**Figure 4:**
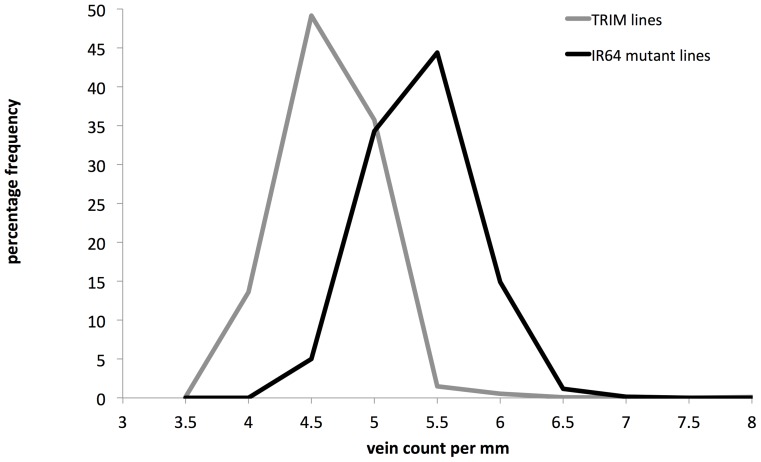
Comparing vein density frequencies of IR64 deletion mutants and Tainung 67 insertion mutants. Frequencies are expressed in percentages of the corresponding mutant population.

Figure 4 compares the frequency distribution of the insertion lines to that of the deletion mutant lines in terms of vein density. Gene activation/disruption created a greater range of vein density values than deletion and point mutations. The increased vein density variation was expected as gene activation was perceived to be a more effective tool in creating phenotypic variation than deletion and point mutagenesis [22] [William P Quick, IRRI, personal communication]. In light of this, vein counts of at least 7 veins mm^−1^ were considered to show an increase in vein density in the insertion mutant population.

There were insertion lines, but no deletion mutants, with vein densities of 3.5 and 8 veins mm^−1^ (0.12% of the insertion mutant population for both vein densities). The deletion mutants peaked at a higher vein count (p<0.001): 5–5.5 veins mm^−1^ compared to 4.5–5 veins mm^−1^ in the insertion lines. This is likely to be attributed to the background genotypes as the Tainung 67 parental line has been consistently shown to have a lower vein density than the IR64 parental line. To support this, in a long term field experiment (data not shown) there was a difference (p<0.001) in vein densities between IR64 wild-type (overall mean of 5.32 ± 0.06 veins mm^−1^) and Tainung 67 wild-type (overall mean of 4.63 ± 0.05 veins mm^−1^). In a long term greenhouse experiment (data not shown), IR64 wild-type had a higher (p<0.001) vein density (overall average of 5.06 ± 0.04 veins mm^−1^) than Tainung 67 wild-type (overall average of 4.77 ± 0.05 veins mm^−1^).

### Inheritance and segregation of vein density mutations

#### Deletion mutant population

Selfing 97 potential deletion candidate lines from the first screening of M_4_ lines with normal leaf width did not always result in progeny with the increased vein density phenotype. Instead, the majority (78%) of mutant lines regressed to the wild-type vein density phenotype in the subsequent generation. Twenty-one M_5_ lines showed transmission of a high vein density trait and did so at varying degrees. Only four M_5_ lines showed high inheritance of the phenotype. However, the progeny of these four lines also reverted to the wild-type phenotype. These lines were abandoned and focus turned to a second screening for narrow width lines.

The second screening of vein densities in deletion mutants with the narrow leaf phenotype used a population constructed by both observations of the original M_4_ screen and previous phenotypic evaluations made by the Genetics Laboratory, IRRI [Marietta Baraoidan, personal communication]. These lines had leaf widths of 4–6 mm for the widest part of the youngest fully developed leaf at the fifth to seventh leaf stage, compared to 8–9 mm for the IR64 wild-type. From 50 narrow leaf lines, 13 were found to have increased vein density progeny. Five lines out of these 13 (E11068-1-10, E19076-1-5, G558-11-5, E22097-1-3 and E26181-1-1) were selected as being candidates after checking for the stability of the higher vein density trait over developing stages, confirming the transmission of the trait to the following generations and analyzing microscopy data.

Segregation data of these lines at the BC_1_F_2_ generation gave ratios that were closer to being Mendelian (1∶3 for a monogenic recessive mutation) (Table 1) than the non-narrow leaf M_5_ deletion candidate mutants. The narrow leaf trait segregated at a 1∶3 ratio for the BC_1_F_2_ lines, E11068-1-10/IR64 and E26181-1-1/IR64 (respectively, χ^2^  =  0.88 and 1.52 < 3.84; df  = 1). The high vein density trait (≥6.5 veins mm^−1^) segregated at a 1∶3 ratio for these two lines, as well as for E19076-1-5/IR64 (respectively, χ^2^  =  0.88, 0.28 and 1.65 < 3.84; df  = 1).

**Table 1:** Number of BC_1_F_2_ progeny of narrow leaf high vein density deletion candidate lines with narrow leaves and high vein densities.

BC_1_F_2_ line	n	n/4	no. progeny with		
			narrow leaves	6.5 veins mm^−1^	veins mm^−1^
E11068-1-10/IR64	152	38.00	43	43	40
E19076-1-5/IR64	182	45.50	34*	38	31*
E22097-1-3/IR64	170	42.50	27**	21***	14***
E26181-1-1/IR64	97	24.25	19	22	14*
G558-11-5/IR64	117	29.25	15**	15**	7***

Narrow leaf width: ≤6 mm. *Observed value differed from expected value (n/4) at p<0.05. **Observed value differed from expected value (n/4) at p<0.01. ***Observed value differed from expected value (n/4) at p<0.001.

Advancing some of these high vein density BC_1_F_2_ progeny did not genetically fix the high vein density. Even though the narrow leafed trait was fixed in BC_1_F_3_ lines, the ratio of high vein density progeny was at best 3∶1 for a monogenic dominant mutation.

A complementation test was performed by inter-mating the candidate mutant lines G558-11-5-2 (♀) and E22097-1-3-1 (♂). The F_1_ progeny had a wild-type phenotype, which suggested that the narrow leafed trait and the high vein density trait in the mutant lines were in separate complementation groups (and so were non-allelic). The F_2_ progeny descended from a single F_1_ parent plant showed a range of phenotypes, some of which were not present in either mutant (or the wild-type) (Table 2). This again suggested independence of the mutations, as well as random assortment.

**Table 2:** Phenotype combinations in the F_2_ progeny of double mutant, G558-11-5-2/E22097-1-3-1.

phenotype	genotype	no. progeny
very narrow leaf & very high vein density	n_1_n_1_n_2_n_2_v_1_v_1_v_2_v_2_	1
very narrow leaf & high vein density	n_1_n_1_n_2_n_2_V_x__v_y_v_y_	2
narrow leaf & very high vein density	n_x_n_x_N_y__v_1_v_1_v_2_v_2_	6
narrow leaf & high vein density	n_x_n_x_N_y__V_x__v_y_v_y_	6
wild-type leaf width & high vein density	N_V_x__v_y_v_y_	14
wild-type leaf width & wild-type vein density	N_V_	50

n_n_: recessive, narrow leaf width allele. N_n_: dominant, wild-type leaf width allele. v_n_: recessive, high vein density allele. V_n_: dominant, wild-type vein density allele. Phenotypes were evaluated at the 5^th^–7^th^ leaf stage except for the vein densities of progeny plants with very narrow leaves. Their vein counts per 2 mm could not be taken at the 5^th^–7^th^ leaf stage so were instead taken at the late vegetative stage. Wild-type leaf width: 8–9 mm. Narrow leaf width: 4–6 mm. Very narrow leaf width: 1–2 mm. Wild-type vein density: ≥5<6.5 veins mm^−1^. High vein density: ≥6.5<7.5 veins mm^−1^. Very high vein density: ≥7.5 veins mm^−1^. Both candidate mutant lines G558-11-5-2 and E22097-1-3-1 had the phenotypic traits of narrow leaves and high vein density. The data characterizes F_2_ progeny descended from a single F_1_ plant. The F_1_ plants all had wild-type vein density and leaf width.

There were three progeny plants with very narrow leaves (1–2 mm leaf width at the fifth to seventh leaf stage), which appeared to demonstrate an additive or synergistic relationship between the narrow leaf mutations of the two mutant parent lines. There appeared to be another additive effect in the case of the two high vein density mutations in seven progeny plants, which had very high vein densities (≥7.5 veins mm^−1^).

In the next round of complementation tests, all possible crossing combinations between candidate lines were attempted. Resultant F_1_ phenotypes gave some indication as to the independence of the mutations. Firstly, the high vein density and narrow leaf width mutations were not necessarily linked. This was most noticeable in the F_1_ plants from E11068-1-10-1/G58-11-5-2 and from E19076-1-5-3/G558-11-5-2. Both crosses generated offspring with wild-type vein density but their leaf widths were narrow (i.e. were of a mutant phenotype) (Table S1).

Secondly, sibling sets of F_1_ plants with wild-type phenotypes (in regard to vein density and/or leaf width) that indicated independence of mutations included E11068-1-10-1 x G58-11-5-2 for vein density, E19076-1-5-3 x E22097-1-3-1 for both vein density and leaf width, possibly E19076-1-5-3 x G558-11-5-2 for vein density and possibly E22097-1-3-1 x G58-11-5-2 for both vein density and leaf width (which would back up the results of the first crossing round presented previously). ‘Possibly’ is relevant in some instances as reciprocal crosses in these mutant combinations gave conflicting results. Disregarding these inconsistencies, it was suggested that the mutations that were independent of each other for vein density were for: E11068-1-10-1 and G558-11-5-2, E19076-1-5-3 and E22097-1-3-1, E19076-1-5-3 and G558-11-5-2, and E22097-1-3-1 and G558-11-5-2.

Given that E11068-1-10-1 and E22097-1-3-1 were considered to share the same mutation for vein density (i.e. their F_1_ progeny had wild-type vein densities), it followed from the independence of E11068-1-10-1 and G558-11-5-2 mutations for vein density that E22097-1-3-1 and G558-11-5-2 vein density mutations should also be independent (Table S1 supports this). From the independence of E19076-1-5-3 and E22097-1-3-1 mutations for vein density and the shared mutation between E11068-1-10-1 and E22097-1-3-1 for vein density, it followed that the vein density mutations for E19076-1-5-3 and E22097-1-3-1 should also be independent. From the independence of E22097-1-3-1 and G558-11-5-2 mutations for vein density and the shared mutation between E11068-1-10-1 and E22097-1-3-1 for vein density, it followed that the vein density mutations for E11068-1-10-1 and G558-11-5-2 should also be independent.

In terms of leaf width mutations, E19076-1-5-3 and E22097-1-3-1 mutations were independent and the mutation of E11068-1-10-1 and E22097-1-3-1 were shared. Therefore, E11068-1-10-1 and E19076-1-5-3 leaf width mutations should also be independent. E22097-1-3-1 and G558-11-5-2 also had independent mutations for leaf width. Given that E11068-1-10-1 and E22097-1-3-1 shared a leaf width mutation, E11068-1-10-1 and G558-11-5-2 should also have independent leaf width mutations, which was contradicted by the experimental findings (i.e. progeny from this cross had narrow leaf widths).

One notable finding from the second round of complementation tests was that all crosses with E26181-1-1-2 resulted in progeny with mutant vein densities and leaf widths.

#### Insertion mutant population

Thirty-five (0.32%) of the insertion lines had increased vein counts per leaf width when screened in Academia Sinica, Taiwan. Twenty-five T_2_ seeds of these lines were grown at IRRI and eight were confirmed for increased vein density. In the T_3_ generation, three lines were identified as potential candidate lines for increased vein density. These three lines, M0104656-B, M0105588-A and M0110124-A, transmitted the increased vein density trait over two generations.

The three candidate lines had a range of vein density values in both the T_2_ and T_3_ generations. Nevertheless, advancing the highest vein density T_2_ progeny to the next generation skewed the values away from the control and towards the upper end of the vein count scale. This was especially evident in the M0105588-A insertion line. This line had only one T_2_ progeny with a higher vein density than the control. However, when this particular progeny was advanced, the majority of its T_3_ progeny had greater vein counts than the control with several (six out of 15) exceeding the threshold for what was considered a candidate. Among these was a progeny plant that averaged 9 veins mm^-1^, which was the highest vein count measured from the screening of both deletion and insertion mutant populations.

The best T_3_ progeny of each candidate line were selected as parents for segregation analysis based upon inheritance data and vein density related phenotypes (Table 3). Segregation ratios were based on using 6.5 veins mm^−1^ as the lower boundary for what was considered to be an increase in vein density, as in the deletion mutant population. M0104656-B-11-5 had a 3∶1 mutant to wild-type vein density ratio (χ^2^  =  1.06 < 3.84; df  = 1) and M0110124-A-2-3 had a 1∶1 ratio (χ^2^  =  0.12 < 3.84; df  = 1). M0105588-A-4-13 did not segregate at a 3∶1 mutant to wild-type vein density ratio by only a very narrow statistical margin (χ^2^  =  3.843 > 3.841; df  = 1).

**Table 3:** Inheritance data for T_4_ insertion candidate lines.

insertion mutant family	vein density segregation			leaf width segregation		
	no. m	no. w-t	ratio	no. m	no. w-t	ratio
M0104656-B-11-5	57	14	3:1	70	1	1:0
M0105588-A-4-13	58	10	3:1*	67	1	1:0
M0110124-A-2-3	38	35	1:1	58	15	3:1

*Observed ratio differed from expected ratio at p<0.05. m: mutant phenotype. wt: wild-type phenotype. The control, Tainung 67 wild-type, had an average of 5.02 ± 0.02 veins mm^−1^ and a maximum of 6 veins mm^−1^. Vein densities of 6.5 veins mm^−1^ were considered to be a mutant phenotype. The Tainung 67 wild-type had two out of eighty-eight plants with <7 mm leaf width, one plant with <6 mm leaf width, and no plants with <5 mm leaf width (and an average leaf width of 9.53 ***±*** 0.08 mm). Leaf widths of 6 mm or less were considered to be a mutant phenotype.

For segregation ratios of leaf width traits, lines M0104656-B-11-5 and M0105588-A-4-13 were fixed (p>0.05 for the line not segregating the expected ratio) for the mutant phenotype (i.e. narrow leaf width) and line M0110124-A-2-3 had a 3∶1 mutant to wild-type ratio (p>0.05 for the lines not segregating at the expected ratio). In M0110124-A-2-3, there were two clear distinct phenotypes for both leaf and plant phenotypes: narrow leaves and dwarfed plant stature compared to wild-type leaf widths and plant stature. Plants with the latter phenotype flowered notably early in comparison to both the mutant segregant phenotype and wild-type Tainung 67. As in the deletion candidate lines, there was evident linkage between the narrow leaf width trait and the high vein density trait in the insertion T_4_ insertion candidate lines. All of the wild-type leaf width progenies in every line had wild-type vein densities.

## Discussion

We have carried out the first study that examines in detail the genetics of leaf vein density in rice, which is considered to be important for the long term goal of inserting the C_4_ mechanism into C_3_ crops. We have shown variation in vein density within a deletion and insertion mutant population, but this was found to be linked to leaf width. Here we discuss the biological nature of the high vein density trait in rice and the significance of our data.

### Evaluating the high throughput vein density screen

The forward genetics scheme was effective in discovering phenotypic alterations relevant to C_4_ photosynthesis. The accuracy of scoring in the screening method for identifying mutant lines with increased vein densities was confirmed by microscopy tests and the consistently higher vein densities of candidate mutant lines in different environments. In addition, the vein density screening established sufficient throughput to identify a workable number of putative deletion candidate mutants over time: average of one candidate line per month. Their number was not so high that there would be too many lines to handle for concurrent in-depth measurements and not so low that there would be no lines taken beyond the screen. The rate of detection was approximately one high vein density event in every thousand lines screened. This frequency of detecting a mutation in leaf anatomy is consistent with that observed in other traits in the mutant collection [13]. Also, this was the same frequency as finding IR64 wild-type plants with a high vein density on one side of the midrib.

The screening used two major assumptions. The first was that the strongest indication of a genetic basis for vein density was trait inheritance. The second was that it was sufficient for a single mutation to have some measurable effect on vein density rather than having to find and combine multiple mutant lines. These two assumptions meant that potentially valuable material had been lost.

The poor inheritance of the high vein density trait in some experiments may have involved an environmental effect on vein density [25]. Different rates of high vein densities were counted in the same line on different measuring sessions (separated by months). The effect of the environment on leaf morphology was demonstrated in [26]. The leaf thickness of rice plants increased if they were transferred from a low light to a high light environment 14 days (but not eight days or less) before full leaf extension [26]. Hence, changes in the environment can alter the morphology of the same leaf number of a rice genotype given a sufficient interval (an acclimation period) between observations.

Variation in leaf morphology as determined by the environment was likely to be an issue in BC_1_F_3_ deletion candidate mutant lines, which should have had a fixed high vein density phenotype after sufficient selfing but instead some of their progeny reverted to wild-type vein densities. The environmental interaction of vein number could have been due to water stress [27], high temperatures [28], and/or variable irradiance, wind and nutrient conditions [29]. This poses an obvious challenge for defining the criteria for the selection of candidates. The environmental effect may indeed have been the cause of the number of false positives in the screening. Both the inheritance of the high vein density phenotype between selfed generations in the narrow leaf lines and the heritability estimate of vein density counters this, however. Narrow sense heritability was reasonably high (h^2^  =  0.56), which meant that most of the variance in the vein density trait was genetic.

### The genetics of high vein density mutations and their link with the narrow leaf width trait

Backcrossing narrow leaf high vein density deletion candidate mutants was performed to investigate the genetics of the two traits of narrow leaf width and high vein density. Results supported the conclusion that the traits were recessive and also indicated that the traits were recessive for a single, and potentially the same, gene. This was to be expected as loss of function mutations by deletion mutagenesis are generally recessive (as the leftover allele can typically produce sufficient protein to compensate).

On the other hand, segregation ratios of T_4_ insertion mutant candidates for increased vein density indicated that the trait was controlled by a single dominant gene. This suggested that gene activations (gain of function mutations) were responsible for the changes in vein density as they behave dominantly [30] [21]. The alternative explanation is that the mutations were caused by somaclonal variation (which occurs at the chromosomal level as in chromosome breakage) [31]. Even though T_4_ line, M0110124-A-2-3 segregated at a 1∶1 ratio for increased vein density, it may still have been due to a monogenic dominant ratio as one of the alleles in the gene locus may have been masking the other one due to allelic dominance.

The narrow leaf trait appeared to be controlled by separate genes in the insertion mutant candidates as it was fixed in the T_4_ lines, M0104656-B-11-5 and M010558-A-4-13 but was only monogenic dominant in T_4_ line, M0110124-A-2-3.

The complementation tests made between deletion candidate mutant lines indicated that the candidate population contained both shared and independent high vein density and narrow leaf width mutations. The tests also showed that the mutation(s) of E26181-1-1-2 is/are key upstream regulator(s) of vein density and leaf width as all progeny sets of crosses that included line E26181-1-1-2 as a parent had mutant phenotypes.

The effort to uncouple the two traits of high vein density and narrow leaf width was difficult. The data seemed to suggest that the high vein density trait could not be inherited independently but was instead a consequence of leaves being narrow. This was what was indicated by [23], which linked the two traits of a high vein density and narrow leaf width by small cell sizes.

The very close overlap between segregation for vein density and for leaf width in the BC_1_F_2_ deletion mutant plants suggested complete linkage between the two traits. However, F_2_ plants from the double mutant, G558-11-5-2/E22097-1-3-1 appeared to uncouple the narrow leaf and high vein density traits. This was evident from progeny plants that had both wild-type leaf widths and high vein densities. Even though this was also found in BC_1_F_2_ plants (as well as in the screening and even the wild-type plants), it occurred at a much higher extent in the F_2_ double mutant progeny: 22% of the progeny plants with wild-type leaf widths had high vein densities compared to <5% in all other mutant families measured. Therefore, even though linkage was present as all the narrow leafed progeny had high vein densities, the linkage was incomplete.

Incomplete linkage was also backed up by the second round of complementation tests. Sets of F_1_ progeny from E11068-1-10-1/G558-11-5-2 and from E19076-1-5-3/G558-11-5-2 had wild-type vein densities but also had mutant leaf widths. This result was in contrast to other genetic results, including the phenotypes of G558-11-5-2/E22097-1-3-1 F_2_ progeny, which suggested that high vein densities followed on from narrow leaf widths in candidate mutants.

### The development of high vein density and the evolution of C_4_ photosynthesis

A series of ecosystem changes took place to bring about the onset of C_4_ evolution. The reduced atmospheric [CO_2_] and other environmental changes associated with open habitats should be regarded as laying down the road, as it were, to C_4_ photosynthesis. The drop in atmospheric [CO_2_] was the precondition that established the early section of the road. However, it was the move of plants out of densely vegetated forests that made them vulnerable to a large host of abiotic and biotic stresses, which first triggered morphological changes within the C_3_ photosynthetic model and then evolved C_4_ photosynthesis [32].

Crucially, changes in the plant's water relations would have required modifications in stomata, venation and leaf dimensions, such as the narrow leaves and high vein densities found in the candidate mutants of this paper. A more efficient hydraulic system is usually present in narrow leaves regardless of changes in vein density (as there is less lamina surface to travel), which is beneficial for superior CO_2_ assimilation rates [33] [34]. In addition, the smaller boundary layer of a narrow leaf also reduces transpiration losses and dissipates excess heat [35], which can be essential in reducing photorespiration losses.

It could be argued that the improved efficiency of CO_2_ utilization and the well-developed leaf vein systems seen in C_4_ systems no longer necessitated flexible alterations in, for example, leaf area, leaf width, angle of leaf blades, leaf thickness and stomatal density seen in C_3_ plants [26] [36].

The link found in this study between narrow leaves and high vein density has been previously reported. In [37], it was explained that leaf shape and venation patterns are closely associated (see also [38]) and that both are probably controlled by a common regulator like auxin. In [39], the intimate link between vein formation and leaf width is likened to fracture propagation in a stretched material, which results in auxin-like signals in the new spaces that form new veins. Therefore, the connection between auxin and vascular formation might explain the connection between vein density and leaf width.

On the other hand, the relationship between leaf shape and venation may depend at least in large part to the underlying cell anatomy. We explore this relationship in our following paper.

## Conclusion

The close linkage of the two traits of high leaf vein density and narrow leaf width was suggested from the results, while the complementation tests showed that it probably was not complete. We conclude that the phenotypically plastic trait of vein density is genetically coupled with leaf width, which itself is a much more genetically robust trait. We hypothesize that a narrow leaf width is a precondition for narrow vein spacing. In the deletion mutant population, a similar finding was found in high biomass mutants, whose high plant height was more genetically robust than increased biomass production [Marietta Barroiden, IRRI, personal communication], which was also found in the evaluation of a range of rice lines in [40]. Therefore, leaf width could be used as a reliable phenotypic marker for finding modifications in vein density, as well as a means of speeding up the throughput of vein density screenings.

The hypothesis that mutagenesis could be used as a tool for creating enough variability latent within the rice genome to uncover measurable increases in leaf vein density was confirmed. The final candidate list included lines containing heritable mutations from all three types of mutagenesis techniques investigated, i.e. point, deletion and insertional mutagenesis. The most significant difference between the techniques was that the former two produced mutations that appeared to be monogenic recessive and the latter produced mutations that appeared to be monogenic dominant.

The study has shown that research in the C_4_ Rice Project can utilize mutant populations for the exploration of a C_4_ relevant trait in rice. A forward genetics scheme is a viable means of gene discovery for other C_4_ traits, given a strong phenotype and a high throughput screening technique. One major advantage of discovering C_4_ anatomy related rice mutants is that they can serve as breeding lines in which the counterpart biochemistry can be inserted. Using increased vein density rice as acceptor lines is especially relevant given that leaf anatomy, and vein density in particular, has been hypothesized to be foundational to the evolution from C_3_ to C_4_ photosynthesis [5] [6] [7].

## Supporting Information

Table S1 **Phenotype combinations in the F_1_ progeny of various crosses between candidate high vein density mutant lines.** Phenotypes were evaluated at the fifth to the seventh leaf stage. Mutant leaf width: 4–6 mm. Wild-type leaf width: 8–9 mm. Mutant vein density: ≥6.5veins mm^−1^. Wild-type vein density: <6.5 veins mm^−1^. A ‘?’ is used where reciprocal crosses gave conflicting results.
(DOCX)
