# Supplementary material for: Increasing Leaf Vein Density by Mutagenesis: Laying the Foundations for C4 Rice
Source: PLoS One. 2014 Apr 23;9(4):e94947. doi: 10.1371/journal.pone.0094947 (PMC3997395; doi:10.1371/journal.pone.0094947)
Supplement: Table S1 — Phenotype combinations in the F1 progeny of various crosses between candidate high vein density mutant lines. Phenotypes were evaluated at the fifth to the seventh leaf stage. Mutant leaf width: 4–6 mm. Wild-type leaf width: 8–9 mm. Mutant vein density: ≥6.5veins mm−1. Wild-type vein density: <6.5 veins mm−1. A ‘?’ is used where reciprocal crosses gave conflicting results. (DOCX) [file pone.0094947.s001.docx]

| **parents** | **mutant**  **no.** | **cross** | **F_1_ phenotype** | |  | **mutations** | |
| --- | --- | --- | --- | --- | --- | --- | --- |
|  |  | **(♀ x ♂)** | **leaf width** | **vein density** |  | **leaf width** | **vein density** |
| E11068-1-10-1 | M1 | M1 x M3 | 4 of 4 plants were mutants | 1 of 4 plants was a mutant |  | M1 = M3 | M1 = M3 |
| E22097-1-3-1 | M3 |  |  |  |  |  |  |
| E11068-1-10-1 | M1 | M1 x M5 | 6 of 6 plants were mutants | all plants were wild-type |  | M1 = M5 | M1 ≠ M5 |
| G558-11-5-2 | M5 |  |  |  |  |  |  |
| E19076-1-5-3 | M2 | M2 x M3 | all plants were wild-type | all plants were wild-type |  | M2 ≠ M3 | M2 ≠ M3 |
| E22097-1-3-1 | M3 | M3 x M2 | all plants were wild-type | all plants were wild-type |  | M2 ≠ M3 | M2 ≠ M3 |
| E19076-1-5-3 | M2 | M2 x M5 | 7 of 7 plants were mutants | 2 of 7 plants were mutants |  | M5 = M2 | ? |
| G558-11-5-2 | M5 | M5 x M2 | 6 of 8 plants were mutants | all plants were wild-type |  | M5 = M2 | M5 ≠ M2 |
| E22097-1-3-1 | M3 | M3 x M5 | all plants were wild-type | all plants were wild-type |  | M3 ≠ M5 | M3 ≠ M5 |
| G558-11-5-2 | M5 | M5 x M3 | 2 of 8 plants were mutants | 1 of 8 plants was a mutant |  | ? | ? |
| E26181-1-1-2 | M4 | M4 x M1 | 11 of 12 plants were mutants | 2 of 12 plants were mutants |  | M4=M1 | M4=M1 |
| E11068-1-10-1 | M1 |  |  |  |  |  |  |
| E26181-1-1-2 | M4 | M4 x M2 | 11of 11 plants were mutants | 1 of 11 plants were mutants |  | M4=M2 | M4=M2 |
| E19076-1-5-3 | M2 |  |  |  |  |  |  |
| E26181-1-1-2 | M4 | M4 x M3 | 10 of 13 plants were mutants | 3 of 13 plants were mutants |  | M4=M3 | M4=M3 |
| E22097-1-3-1 | M3 |  |  |  |  |  |  |
| E26181-1-1-2 | M4 | M4 x M5 | 5 of 5 plants were mutants | 2 of 5 plants were mutants |  | M4=M5 | M4=M5 |
| G558-11-5-2 | M5 |  |  |  |  |  |  |
